# Biology of *Chiloloba orientalis*

**DOI:** 10.1673/031.012.12701

**Published:** 2012-11-03

**Authors:** S.M. Kumbhar, A.B. Mamlavya, S.J. Patil, G.P. Bhawane

**Affiliations:** Department of Zoology, Shivaji University, Kolhapur, Maharashtra, India

**Keywords:** beetles, Cetoniinae, emergence, incidence, waste recycling

## Abstract

This study, related to emergence of the cetoniid beetle, *Chiloloba orientalis* D and R (Coleoptera: Scarabaeidae: Cetoniinae), was conducted annually from August to the middle of October from 2007 to 2010 in maize (*Zea mays* L. (Poales: Poaceae)), sorghum (*Sorghum bicolor* (L.) Moench), and grass (*Hetropogon contortus* (L.) P. Beauv. ex Roem. and Schult., *Apluda mutica* L.) cultivated fields in a selected plot of grassland in Kolhapur, Maharashtra, India. Adults of *C. orientalis* feed on the inflorescence of *S. bicolor*, *Z. mays*, *H. contortus* and *A. mutica.* The occurrence of adults in this study indicates that the emergence of beetles coincides with the flowering period of its host plants. The immature stages of this beetle feed on the decaying organic matter of crop residues in composting heaps, indicating these immature stages play a key role in the recycling of organic waste of plant and animal origin and help in the enrichment of soil nutrients, especially in the red brown soil where primary decomposers are scarce. This study provides detailed information on the morphological peculiarities of immature stages with the duration required for the completion of the life cycle. The average incubation period of eggs was 15.2 days. The first, second, and third instar lasted for 22.7, 54.3, and 46.6 days respectively. The mean pupal period was 14.7 days. The average adult longevity was 9.4 days.

## Introduction

The subfamily Cetoniinae (Coleoptera: Scarabaedae) is a diverse group of scarabs, comprising approximately 3900 species in 315 genera (Mico et al. 2008). Cetoniids are distributed worldwide except for sub-polar areas and some offshore New Zealand Islands. Cetoniinae is not only comprised of shiny, bright, metallic species, but also velvety forms with cryptic disruption patterns.

Adults of most species exhibit diurnal feeding habits, feeding on flowers as well as plant saps and fruits. There are few explicit descriptions of predacious cetoniids. Ghorpade (1975) reported a cetoniid, *Spilophorus maculatus*, that fed on the nymphs of the tree hopper, *Oxyrhachis tarandus*, which is a pest of *Acacia concinna.* Among phytophagous cetoniinae, mouth-parts are adapted for dealing with soft food, and labrum are membranous and concealed; generally, mandibles are thin and not capable of biting, and the maxillae are invested with long hairs.

Orozco and Pardo-Locarno (2004) reported that around 61 cetoniid species are known with the larval descriptions. Mico et al. (2000) gave new larval descriptions of two species of *Euphoria.* Some reports were available on the distribution, development, description of immature stages, and conservation of the lesser known cetoniids all over the world (Ratcliffe 1976; Woodruff 1982; Domeck and Johnson 1988; Peck and Thomas 1998; Smith, 2003).

Deshpande and Rao (1980) provided four new records of cetoniid beetles from the state of Maharashtra in India (*Anatona stillata*, *Anthracophora crucifiera*, *Oxycetonia versicolor*, *Chiloloba orientalis*). *C.* *orientalis* plays an important role in the pollination of *Sorghum bicolor* (L.) Moench (Poales: Poaceae), *Zea mays* L., and *Pennisetum typoides.* No discussion was previously available about developing stages of *C. orientalis.* The objective of the present effort is to study the incidence of *C. orientalis*, its life cycle, and the structural peculiarities of its immature stages.

## Materials and Methods

### Experimental Site

**Cultivated fields of maize and sorhgum.** The observations on the adult emergence were recorded in the maize and sorghum cultivated areas near Kolhapur, Maharashtra, India. The area of observation for maize and sorghum was one hectare. The observations were carried out at three different localities.

**Grassland**. The observations on the adult emergence were also recorded in the grassland at the Kolhapur district. The grasses selected for the observations were *Heterpogon contortus* (L.) P. Beauv. ex Roem. and Schult. (Poales: Poaceae) and *Apluda mutica* L. The observations were carried out at four different sites. The observations at each site were carried out in a 10 × 10 m plot at Shivaji University, Kolhapur, Maharashtra, India.

**Observation on the beetle emergence.** The observations were conducted weekly for four years (2007 to 2010) from August to the middle of October. The beetle emergence was recorded before, during, and after the flowering of host plants.

### Density and collection of immature stages and beetles

**Grubs**. The grubs were counted in quadrates with an area of 10 square feet, at 25 to 30 cm depth, at three random places in each dung manure bed. Ten such observations were taken randomly out of 20 different dung manure beds from September to January on a fortnightly basis.

**Beetles**. The number of beetles on the four selected host plants were counted weekly from the first day of emergence of adults. This observation was based on the settling of beetles on the cobs and spikelets of the host plants. Mean data expressed the density of beetles per plot of host plants. As the beetles are diurnal in habit, the collection of beetles was carried out during morning hours, from 07:30 to 10:00.

### Laboratory rearing of beetles for life cycle study

Beetles were collected from fields of *S. bicolor* and *Z. mays* around Kolhapur, Maharashtra, India. Ten beetles of each sex were released in earthen pots and maintained in the laboratory at 25° C to 30° C with a 12 hour day length and relative humidity varying from 65% to 75%. The beetles were fed on fresh, tender cobs of Sorghum. Earthen pots were half-filled with a mixture of moist sand and soil in equal proportion. Tap water was added to keep the soil damp. The mouths of the pots were covered with muslin cloth to prevent beetles from escaping.

Twice a week, soil was tipped out of pots, and eggs were removed and placed on moistened filter paper in 90 mm petri dishes, 35 to 40 eggs per dish. The same soil was used for about a month, and was examined every time it was tipped out of the pot. Petri dishes with eggs were kept in the dark inside the moist earthen pot. Hatched larvae were removed, and the paper was kept moistened. Newly hatched larvae survived well in the petri dishes by eating the damp filter paper. Twenty newly hatched larvae were placed in each earthen pot (30 cm depth and 27 cm upper diameter) half-filled with moist sand and a soil mixture in which compost was added as a food for larvae. This medium was replaced every two weeks.

Larvae pupate in earthen cells were removed and placed in earthen pots containing a moist mixture of sand and soil and covered with muslin cloth. Up to 50 pupae were placed in each pot. The sand and soil mixture in the pots was moistened twice a week with tap water until the emergence of beetles. All the measurements of developing stages were made by millimetric paper and put to statistical analysis.

Fecundity observations were made by releasing a pair of beetles separately in moist sand and soil mixture in earthen pots as described earlier. The number of eggs laid by 10 females was recorded.

### Results

#### Emergence of *C. orientalis*

An emergence of *C. orientalis* coincided with the flowering period of Maize, Sorghum, *H. contortus* and *A. mutica.* The field observations on the occurrence clearly showed the adults emerged during the first week of September and continued up to the middle of October. Beetles fed on the floral parts of the grasses, both cultivated and uncultivated species. Cultivated species were *Z. mays* and *S. bicolor.* The wild species, which were found in abundance in the grassland, were *H. contortus* and *A. mutica.* The emergence of beetles coincided with the flowering period of maize, sorghum, *H. contortus* and *A. mutica*

**Figure 1.**
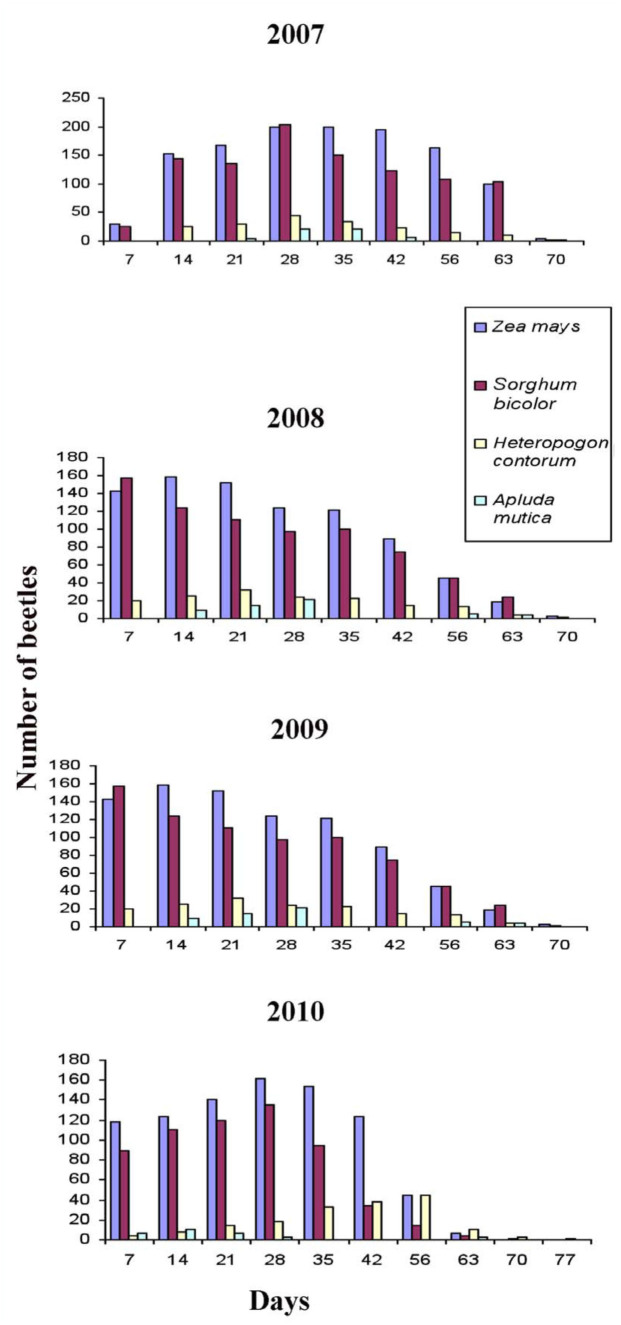
Incidence of *Chiloloba orientalis* on four different host plants during August to October in four consecutive years (2007 to 2010). High quality figures are available online.

The beetle showed species specificity with respect to host plant selection for feeding and mating. In the maize and sorghum cultivated fields, the maximum number of individuals was recorded on maize followed by sorghum. Where maize and sorghum were not available, such as in the grassland, beetles fed on the spikelets of *H. contortus* followed by *A. mutica.* The incidence of *C. orientalis* on four different host plants from August to the middle of October 2007–2010 is shown in Figure 1.

#### Density of grubs within dung manure beds

**Figure 2.**
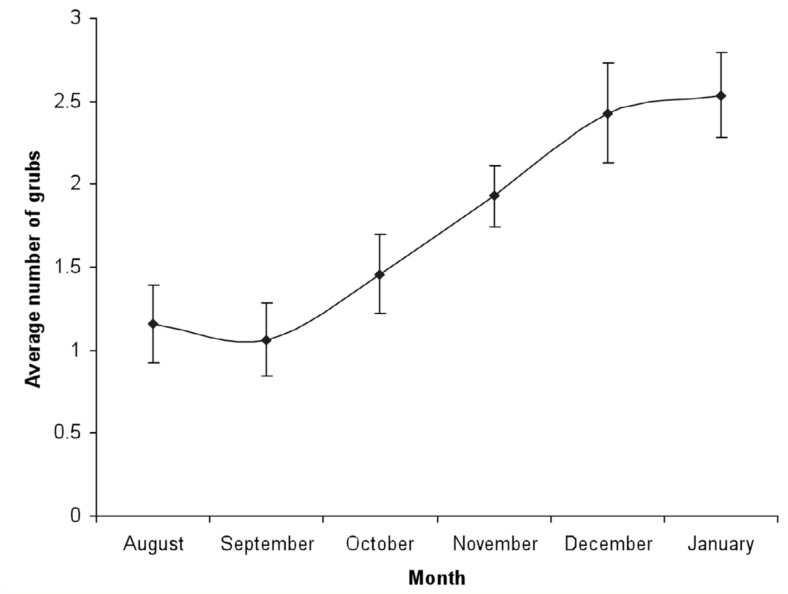
Average density of *Chiloloba orientalis* in dung manure beds during 2007 – 2010. High quality figures are available online.

The mated females laid eggs within dung manure beds at a depth of 15 to 20 cm from the surface of the bed. After hatching, the grubs fed greedily on the decaying dung and plant material within the dung manure bed. Figure 2 shows the density of grubs within the prepared dung manure beds. Usually, the area of the dung manure bed was 10 square feet. The occurrence of grubs was recorded from September to January.

#### Life cycle of *C. orientalis*

**Figure 3.**
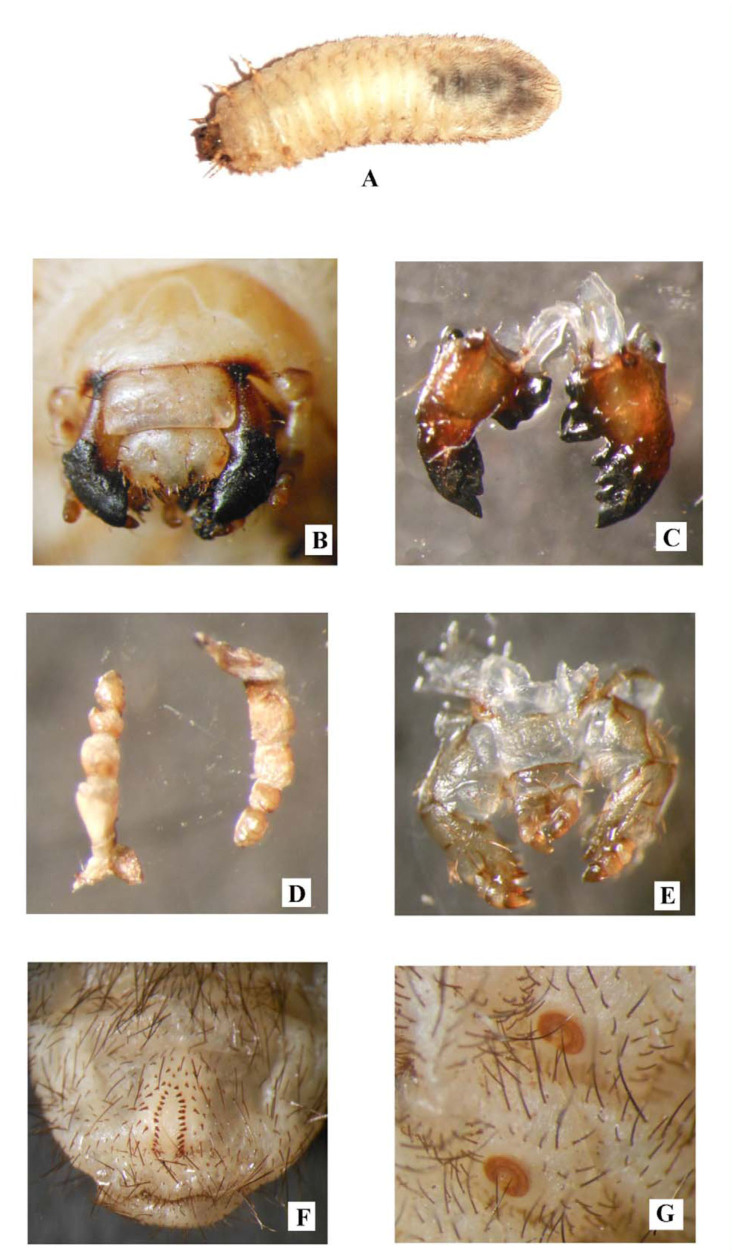
A) Third instar larva of *Chiloloba orientalis.* B) Frontal view of head capsule. C) Dorsal view of mandibles. D) Dorsal and ventral view of antennae. E) Dorsal view of maxillae. F) Venter of last abdominal segment showing details of pallidium. G) View of “C” Shaped spiracle. High quality figures are available online.

**Figure 4.**
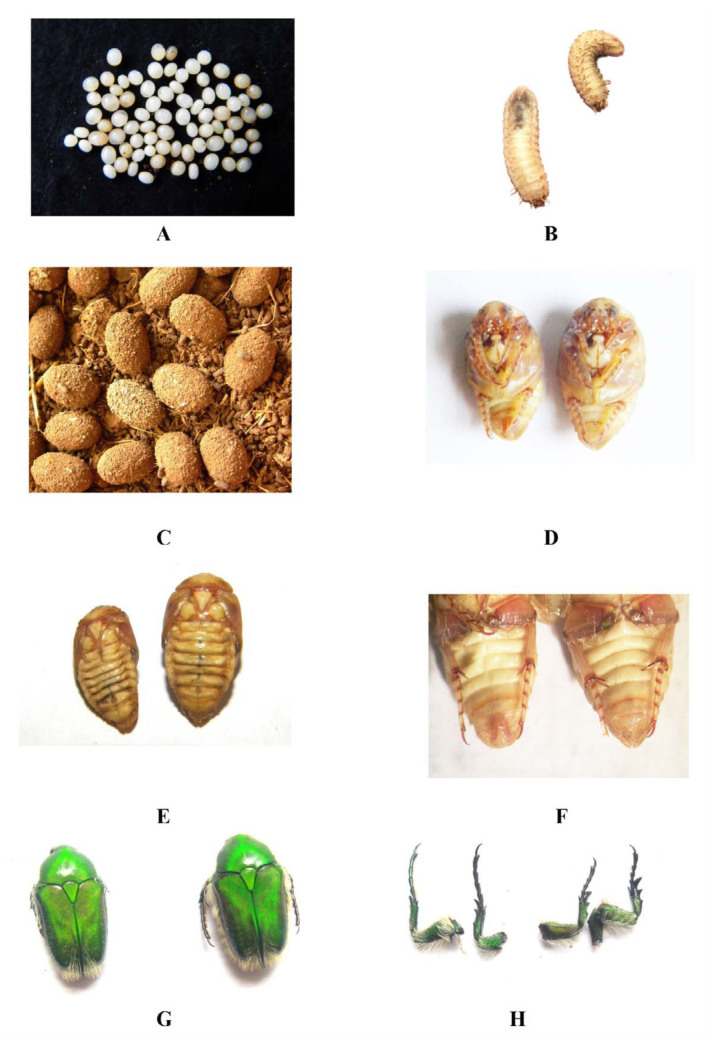
Biology of *Chiloloba orientalis.* A) Eggs. B) Ventral view of second third instar. C) Pupal chambers prepared by the third instar larvae from decomposing dung for pupation. D) Pupa: male and female ventral view. E) Pupa: male and female dorsal veiw. F) Ventral view of male and female pupae showing the slender, tapering, pointed tibial spurs of the male and the broad, flattened, blade-like spurs of the female. G) Dorsal view of adult male and female. H) Front tarsi of male and female. Front tarsi in male are longer than the female. High quality figures are available online.

The details of the developing stages are presented in Tables 1 and 2. Details of the fecundity studies are presented in the Table 3. The immature stages and sex dimorphic characters of pupae and adults are shown in Figure 3 A–H. The morphological peculiarities of third instar are presented in Figure 4 A–G.

#### Eggs

Field observations on mating indicated that it occurs in August to September. Mated females laid eggs in soil and sand mixture in the pots at a depth of 4 to 6 cm. Fecundity observations under laboratory conditions revealed 10 mated females laid an average of 73.1 eggs, ranging from 69 to 75 eggs per mated female. Freshly laid eggs were dirtywhite in color and oval in shape. The length of egg ranged from 2 to 4 mm with a mean of 2.4 mm, while the width ranged from 1 to 2 mm with a mean of 1.3 mm. The incubation period ranged from 11 to 18 days with a mean of 15.2 days.

#### First Instar

Body dull-white in color, the head capsule of newly hatched larvae light-brown in color and became dark-brown as the instar progressed. The duration of first instar ranged from 20 to 25 days with a mean of 22.7 days. The length of fully grown larvae ranged from 4 to 6 mm with a mean of 5 mm, while the width ranged from 2 to 3 mm with a mean of 2.4 mm.

#### Second Instar

The larvae were structurally similar to first instars except in size. The length of second instar ranged from 14 to 17 mm with a mean of 15.5 mm, while the breadth ranged from 3 to 5 mm with a mean of 3.6 mm. The duration of second instar ranged from 45 to 60 days with a mean of 54.3 days.

#### Third Instar

Body cylindrical, dull-white in color, typically scarabaeform, head capsule width 3 mm. Spiracles distinct, ‘C’ shaped. The length of ten full-grown third instar larvae ranged from 22 to 26 mm with an average 23.1 mm, while the breadth ranged from 5 to 7 mm with an average 5.4 mm. The duration of third instar ranged from 45 to 50 days with a mean of 46.6 days.

Cranium yellowish-brown, epicranial suture a depression, frontal suture distinct with precoilae, epicranial surface wrinkled with setae forming a row of two dorsoepicranial setae with 6 or 7 smaller setae, and with one pair of setae on lateral edges. Oceli absent. Epicranium and frons with pores. Posterior part of the clypeus more sclerotized, anterior clypeal setae long, paramedical, and close to the proximal end of clypeus. Pair of exterior clypeal setae on lateral edges. Clypeus with pores. Labrum rounded at margins. Pores present. One pair of long setae centrally, three pairs of long setae (one short and two long) proximally. Three pairs of long setae on lateral sides. Apical lobe with fringe of setae. Antenna with four unequal antennomeres. Antennomeres devoid of setae, antennomere I longer than others. Antennomere IV bears three dorsal and one lateral oval shaped sensory spots. Mandibles prominent left mandible with three cutting edges and right mandible with two cutting edges, one long setae at the side of scissorial area. Stridulatory area visible on ventral side. Molar parts of both mandibles with distinct lobes. Cardo of maxillae with short setae, maxillary palp with three segments, ventral side of the stipes with few long setae. Dorsolateral edge with three teeth (one long and two short). Labium with a pair of long setae, prementum of labium with few long setae, glossa covered with short and long setae. Epipharynx with rounded lateral margins. Spiracle prothoracic, legs light in color and weak. Legs covered with short and long setae, claw small and nearly blunt. Spiracles on abdominal segment 1 to 8, covered with rows of long and short setae on dorsal and ventral surface. Septula present extending from lower anal lip to venter of tenth abdominal segment. Tigilla covered with short sharp setae. Barbulae with long setae, anal opening transverse and straight surrounded by rows of long and short setae.

#### Pupa

Full-grown larvae moved down in earthen pots and ceased feeding. Larvae constructed small earthen cells and pupated inside. Pupal chamber oval in shape, length 14 mm, width 10 mm (n = 10), composed of faecal pellets. It is rough externally and smooth-walled internally, with final instar exuvium closely applied inside. Pupa exarate. Head reflexed beneath pronotum, cranium shiny. Clypeus elongate with deep groove anteriorly, emarginate. Antennae 9 segmented, segment 1 longer than 2–4, segment 5 and 6 smaller in size, and segment 7–9 lamellate. Segments 1–3 beared thin, long, setae. Scape beared long thin setae on anterior side, setae short, mid-dorsal on pedicel, fringe of setae on inner lateral side of scape. Maxillary palpi four-segmented, terminal segment longer, labial palps three-segmented, segment 1 and 2 short and segment 3 longer, mentum with setae. Pronotum well developed, smooth, shiny, mesonotum, dominated by subtriangular, scutellum, metanotum depressed where scutellum projects over it. Elytra curled beneath the abdomen. Hind wings membranous, obscured by elytra. Coxae of hind leg large, femora long and dorsoventrally flattened, tibae terminating with spurs, tarsi five-segmented, terminal segment longer. Claws sharp, bifid, devoid of empodium. In pupa, sex dimorphic characters were present, male pupa showed separate eighth and ninth segment with median elevated lobe-like area representing the attachment of adeagus, female pupa absent. The length of 10 pupae ranged from 13 to 14 mm with an average 13.2 mm, while the breadth ranged from 7 to 9 mm with an average 8 mm. The pupal period lasted 14 to 15 days with an average 14.7 days.

#### Adult

Newly hatched adults were found in earthen cells in February. Adults remained in the pupal cells for seven months, and their appearance was continued after mid-August until the end of October. One year was required for the completion of the life cycle. Adults were bright metallic green in color and covered with yellow hairs above and beneath. The length of adult ranged from 12 to 15 mm with a mean of 14 mm, while the width ranged from 7 to 9 mm with a mean of 8.7 mm. The adult longevity ranged from 8 to 10 days with a mean of 9.4 days. Eyes prominent. Prothorax narrower at posterior end with base deeply excised in middle. The sides were slightly opaque. Scutellum long and narrow, having concave sides and sub acute apex. Elytra deeply sinuated at base. Inner margins of elytra elevated posteriorly and developed into sharp spines at apices. Sternal process flat and transverse; front tibiae tridentate. Two terminal teeth of the front tibiae long and sharp. Pygidium covered with long hairs. Front tarsi longer in male than in the female. Terminal spines of elytra prominent in males compared to females. In males, the hind leg had three tibial spurs at its distal end along its inner margin. The misai spur was slender, tapering and pointed at apex. The hind legs of females also had three tibial spurs, but the spur at the misai end was broad, flat, and sufficiently strong and blade-like.

### Discussion

From the field and laboratory data, certain inferences about the incidence, food plants of adults, and life cycle of *C. orientalis* were being made.

The emergence of adults coincided with the flowering period of its food plants. Adults fed on the inflorescence of graminacius plants, which include both cultivated and undomesticated species. *Z. mays* and *S. bicolor* are cultivated species and both form the staple food for many cultures. *H. contortus* and *A. mutica* are undomesticated species and occur abundantly in the grasslands. The emergence of most of the scarab beetles depended on the soil moisture, temperature, and availability of food. The metallic green beetle, *Chiloloba acuta*, was reported to feed on sorghum pollen grains (Ayyar 1963). *Polystigma punctata* is frequently recorded on the flowers on *Bolama spinosa* in certain areas of New South Wales (Howkeswood 1990; Howkeswood 2003).

The observations on the selection of food plants revealed that beetles showed specificity in selection of food plants according to their availability. The primary host plants of *C. orientalis* were *S. bicolor* and *Z. mays* in cultivated area. In the absence of these species, beetles fed on the spikelets of *H. contortus* and *A. mutica* in the grasslands. Ratcliffe (1991) noted that adults of *Euphoria sepulcralis* fed on tree sap, a wide variety of ripening fruits, corn, flowers of apple, thistle, mock orange, milkweed, dogwood, sumac, yarrow, daisies, and golden rod. The beetles of *E. sepulcralis* were found associated with 126 different plant species.

The mating of *C. orientalis* occured on the inflorescence of host plants. Mated females laid eggs within the artificially prepared buffalo-dung manure beds at a depth of 20 to 25 cm. The embryo became visible prior to hatching from the egg. The mated females of *Protaetia fusca* (Herbst) (Coleoptera: Scarabaeidae: Cetoniinae) laid eggs in the cotton mulch. The eggs of *P. fusca* were oval in shape, the average length of egg was found to be 2.2 mm, and the maximum width was 1.6 mm. Chorion were whitish in color, smooth, and glossy. The average incubation period of egg was nine days. The abdominal segments of the embryo were able to be distinguished through the chorion as development proceeded (Simpson 1990). Veeresh and Veena Kumari (1985) studied the feeding and breeding behavior of lamellicorn beetles and reported that a number of species of scarabaeid beetles remained near to the anus of sloths and monkeys in order to oviposit on their dung. In *P. fusca*, the oviposition occured only when suitable substrate was available. The laboratory observations revealed that females did not oviposit on a tissue paper replacement and eggs were reabsorbed (Simpson 1990). *Netocia* species lay their eggs in rich organic soil close to heaps of rabbit dung and heaps of remains close to ant nests (Donaldson 1979).

The larvae were typically scarabaeform, phytosaprophagus, or xilosaprophagus, and passed through three larval stages before they completed their metamorphosis into pupa (Dutto 2005, 2007; Razov et al. 2009) The grubs of *C.* *orientalis* fed on the decayed plant material in the composting pit rather than the freshly added dung within dung manure beds. The immature stages of cetoniids usually fed on the organic matter in the soil, rotten wood, trash, and other accumulated debris within hollows of trees (Ritcher 1958; Simpson 1990; Sipek 2009). The food preference in immature stages of chafer beetles was species specific. *P. fusca* larvae occured commonly within the humus. The larval stage lasted for 39 weeks (Simpson 1990). It was very common for immature stages of cetoniid beetles to occur in decaying wood and vegetable matter. Mico and Galante (2003) reported feeding habits and biology of four cetoniids. The immature stages of *Potocia cuprea*, *Potocia opaca*, and *Netocia* species were found in organic substrates such as vegetable matter within the soil around the roots of plants, ant nests, rabbit latrines, and wood. The immature stages of *P. cuprea* and *P. opaca* consumed the decayed wood of *Ficus carica*, *Ceratonia siliqua*, and *Phoenix dactilifera*, and played a crucial role in the decomposition of wood. There are certain species whose developing stages fed on the cattle excrements. The grubs of *Pachnoda sinuata flaviventris* were commonly found in composting heaps (Donaldson 1979). The larvae of *Euphoria sepulcralis* were found in soil beneath dead sod or manure (Ritcher 1945).

The grubs of *C. orientalis* feed only on the decaying organic matter in the dung heaps, but the larvae were not recorded within the dung pads in pastures. In pastures, the grubs were found in composting heaps. From this observation, it seems that females laid eggs where a sufficient quantity of food was available to young ones for the entire period of development. This observation indicates that the suitable site with ample food is selected by the parent for the entire development of young ones. Dutto (2005, 2007) reported that females laid eggs in the substratum rich with organic matter of rotten vegetation origin. Before pupation, the grubs of *C. orientalis* move deep into the composting manure, prepare earthen cells, and pupate within the cells. Larvae of *P. punctata* develop in the ground and pupate in an earthen cell (Howkeswood 2003). The fullgrown grubs of *P. fusca* construct the pupal chamber within the humus (Simpson 1990). The pupa of *P. fusca* lasted four weeks (Simpson 1990). The last instar larva of *E. sepulcralis* prepared an earthen cell and pupated within the cell (Hayes 1925). The length of the pupal stage of *E.* *sepulcralis* averaged 15.4 days (Ratcliffe 1991).

The biology of cetoniids differs and depends on the species. Some species complete their life cycle in a few months, while others require more than a year. *C. orientalis* had a life cycle of one year. Mico and Galante (2003) reported that *Netocia oblonga* had a life cycle of one to two years. Ligima and Takeuchi (2007) studied the life history of *Protaetia orientalis* and reported that it required one year for the completion of one generation. Simpson (1990) studied the biology of *P. fusca* and reported that it required about 44 weeks for completion of one generation. *E. sepulcralis* had a life cycle of one year (Ritcher 1945).

The larvae of Scarabaeid beetles either cooperate in soil processes or live in the soil at some stage in their development (Kuhnelt 1976). Though some species are strictly predatory in nature or feed on carrion, a wide range of beetles and their immature stages live in fresh or decomposing vegetable matter on or in the soil (Raw 1967). The grubs of the family Scarabaeidae are completely herbivorous or saprophagous (Raw 1967; Crowson 1981). Coprophilous beetles of the subfamily Scarabaeinae are actively engaged in organic transformation, particularly in grassland ecosystems. The individuals of this group feed on animal excrements and debris in the soil and on grass roots (Carne 1956).

The immature stages of the subfamily Cetoniinae are known as very active digesters of organic material in the soil. The larvae of cetoniid beetles feed on the organic matter, which mixes the organic and inorganic material and redeposits them in the form of cylindrical pieces of excrement. In this way, grubs turn over the soil and enrich it with organic matter (Kunhelt 2007). *C. orientalis* plays an important role as an adult as well as in larval form. Adults assist in the pollination of *S. bicolor* and *Z. mays* in the cultivated fields of these crops, which may result in an increase in yield of these staple foods. Grubs are actively involved in the decomposition of organic waste of plant and animal origin. In the future, efforts will be made to utilize *C. orientalis* grubs in organic waste recycling.

**Table 1.**
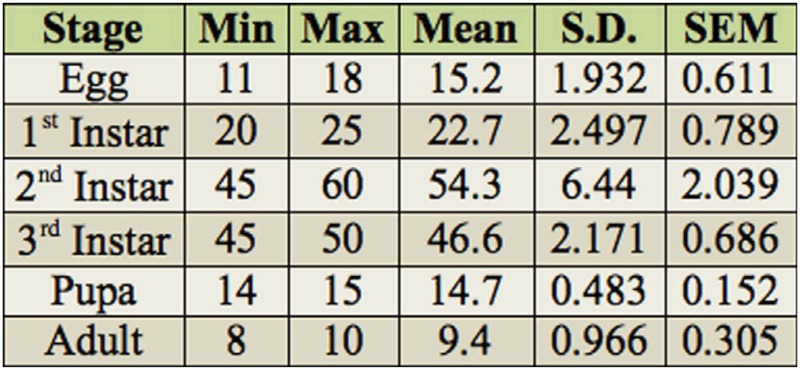
Duration of different stages of *Chiloba orientalis.*

**Table 2.**
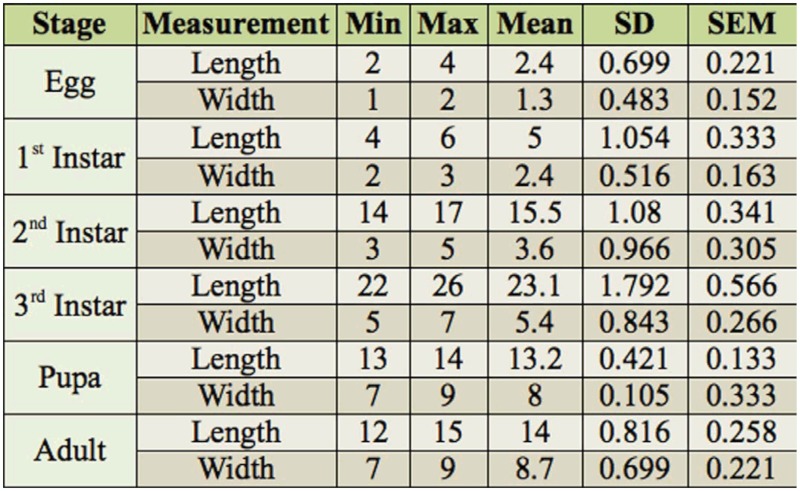
Morphometric data of *Chiloba orientalis.*

**Table 3.**
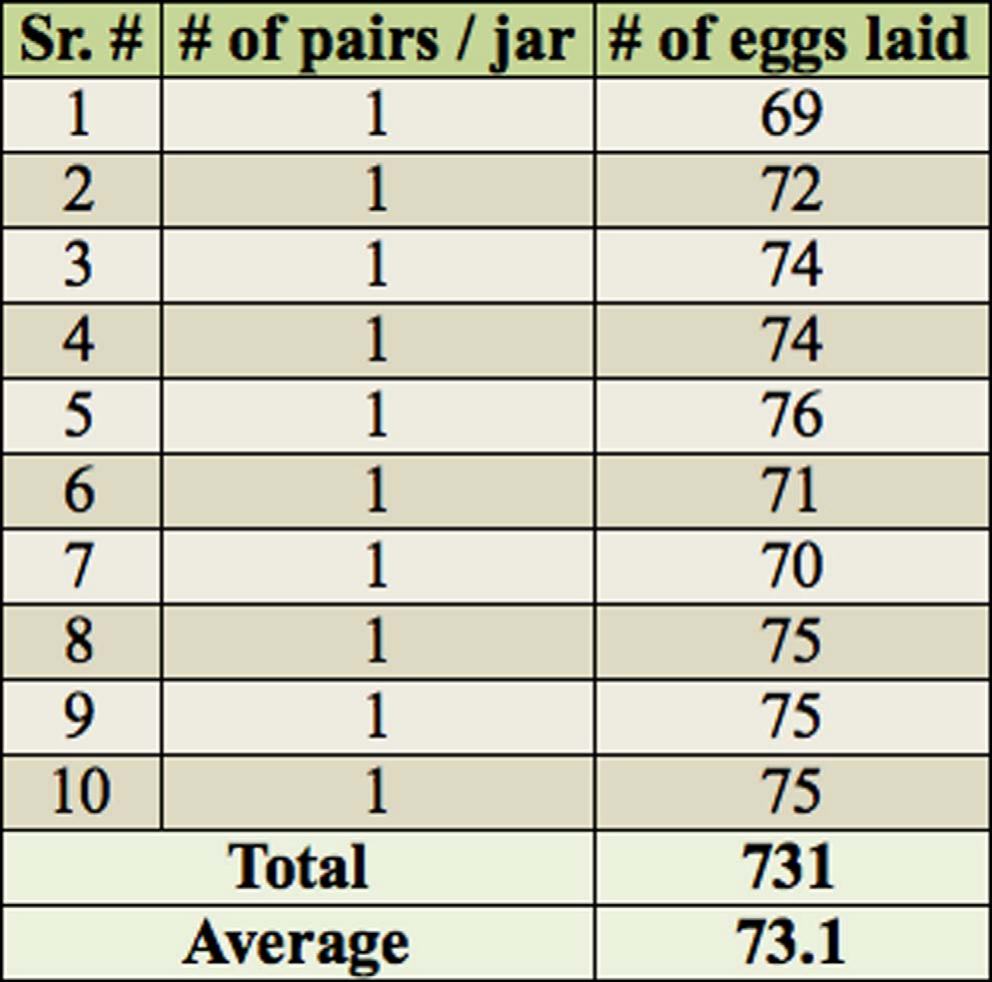
Fecundity of *Chiloba orientalis* recorded under laboratory conditions.
